# The First Open Fetal Surgery for Myelomeningocele Repair in a Bicornuate Uterus With Anterior Placenta in Central America: A Case Report From Nicaragua

**DOI:** 10.7759/cureus.117452

**Published:** 2026-10-04

**Authors:** Alma Celeste Avilés Castillo, María Esther Suárez García, José Luis Rodríguez, Rodrigo Gonzalez, Oswaldo José Pérez Vargas, Marcela Conrado Chamorro, José Luis Villavicencio Bermudez, Jasser Logo Cabrebra, Jehmis Guevara Guevara, Christopher Kaleb Romero Ríos

**Affiliations:** 1 Obstetrics and Gynecology, Hospital Militar Escuela "Dr. Alejandro Dávila Bolaños", Managua, NIC; 2 Neurosurgery, Hospital Militar Escuela "Dr. Alejandro Dávila Bolaños", Managua, NIC; 3 Anesthesia, Hospital Militar Escuela "Dr. Alejandro Dávila Bolaños", Managua, NIC; 4 Research, Hospital Militar Escuela "Dr. Alejandro Dávila Bolaños", Managua, NIC

**Keywords:** bicornuate uterus, fetal surgery, myelomeningocele, neural tube defect, prenatal repair, spina bifida

## Abstract

Open fetal surgery for myelomeningocele (MMC) has shown superior outcomes compared with postnatal repair, but it remains unavailable in most low- and middle-income countries (LMICs). We report the first open fetal MMC repair performed in Central America, in a patient with a bicornuate uterus, anterior placenta, and a gestational age beyond conventional criteria. A 27-year-old woman (BMI: 28.6 kg/m²) with epilepsy treated with carbamazepine and late prenatal care was referred at 26 5/7 weeks for a fetus with lumbosacral MMC, lemon sign, bilateral ventriculomegaly (10.5 mm), and an obliterated posterior fossa. A bicornuate uterus with an anterior placenta was identified. After multidisciplinary evaluation involving Maternal-Fetal Medicine, Fetal Surgery, Pediatric Neurosurgery, Neonatology, and Anesthesiology, open fetal microneurosurgery was performed at 27 5/7 weeks using microsurgical myeloplasty with continuous intraoperative Doppler monitoring.

Postoperative surveillance showed stable ventriculomegaly, regression of posterior fossa herniation, and an intact repair site. Cesarean delivery at 33 weeks was performed for severe oligohydramnios secondary to premature rupture of membranes, resulting in the birth of a 2,060 g male with Apgar scores of 8/9 and intact lumbar closure. The neonate required eight days in the neonatal intensive care unit (NICU) and did not require cerebrospinal fluid (CSF) diversion. Follow-up to postnatal day 47 confirmed a favorable neurological outcome. Open fetal MMC repair was feasible in an LMIC hospital with appropriate multidisciplinary coordination, even in the presence of a bicornuate uterus with anterior placenta and a gestational age beyond conventional criteria. During the documented follow-up period, the neonate showed favorable short-term neurological outcomes without the need for CSF diversion.

## Introduction

Myelomeningocele (MMC) is the most severe form of open neural tube defects (NTDs), causing motor impairment, hydrocephalus, and Arnold-Chiari type II malformation [1]. The Management of Myelomeningocele Study (MOMS) randomized trial showed that prenatal repair before 26 weeks of gestation reduced the need for ventriculoperitoneal shunting from 82% to 40% and improved motor outcomes at 30 months compared with postnatal surgery [1,2]. Eligibility criteria for the MOMS trial included a gestational age between 19 0/7 and 25 6/7 weeks, MMC with an upper boundary at T1 through S1, evidence of hindbrain herniation, a normal karyotype, a singleton pregnancy, and a BMI of 35 kg/m² or less [1].

However, access to fetal surgery remains limited in low- and middle-income countries (LMICs), where NTD prevalence is paradoxically highest because of insufficient folic acid supplementation and limited prenatal screening [3]. In Latin America, Chile and Mexico have contributed to an international consortium that has reported outcomes from more than 300 fetoscopic cases [4]. Brazil has accumulated the largest experience with both open and fetoscopic techniques [5], while Argentina has reported outcomes with open fetal MMC repair, including cases beyond the conventional 26-week gestational age window [6].

Two principal techniques exist for prenatal MMC repair: open hysterotomy, as used in the MOMS trial, and fetoscopic approaches that aim to reduce uterine morbidity by using smaller access ports [5,7]. While both approaches aim to cover the exposed neural placode, open repair remains the most widely validated technique; fetoscopic repair, although promising, is still considered investigational at many centers [7]. The open approach carries well-documented maternal risks, including higher rates of blood transfusion, pulmonary edema, oligohydramnios, chorioamniotic membrane separation, and uterine dehiscence or rupture at subsequent delivery [3].

Open fetal MMC repair in the setting of uterine malformations, such as a bicornuate uterus with an anterior placenta, poses additional technical challenges, including a restricted operative field and limited hysterotomy site options. To our knowledge, no prior case of prenatal MMC repair in a bicornuate uterus has been reported in the literature. We identified this gap through a targeted search of PubMed, Scopus, and Google Scholar using the terms "fetal surgery," "myelomeningocele," "bicornuate uterus," and "uterine malformation," with no language or date restriction, which yielded no relevant results. We present the first reported open fetal microneurosurgery for MMC performed in Central America, in a patient with a bicornuate uterus, anterior placenta, and a gestational age of 27 5/7 weeks at surgery, beyond the gestational age window established by the MOMS trial [1]. This report follows the CARE guidelines for case reports.

## Case presentation

A 27-year-old woman, gravida 3 para 0 (one prior low transverse cesarean delivery of the Munro-Kerr type, one spontaneous abortion), BMI 28.6 kg/m², was referred at 26 5/7 weeks of gestation after late initiation of prenatal care (first contact at 20 weeks). Her history was notable for epilepsy diagnosed at age three, managed with carbamazepine 400 mg daily (seizure-free for four years). No periconceptional folic acid supplementation was documented. Genetic testing was not available at our institution. She was hemodynamically stable with a fundal height of 24 cm, a fetal heart rate of 145 to 150 bpm, no uterine activity, and a closed cervix.

Neurosonography revealed a lemon-shaped calvarium and bilateral ventriculomegaly (10.5 mm) (Figure 1). Evaluation of the posterior fossa showed an obliterated posterior fossa with a banana-shaped cerebellum, consistent with Arnold-Chiari II malformation (Figure 2A). A cystic lesion containing nerve roots was identified at L5, with a vertebral opening extending from L5 through S3 and a functional level of S1-S2, based on observed ankle and knee flexion without equinovarus (Figure 3). Three-dimensional ultrasound rendering of the myelomeningocele sac yielded an estimated volume of 2.0 cm³, calculated from orthogonal linear dimensions (Figure 4).

**Figure 1:**
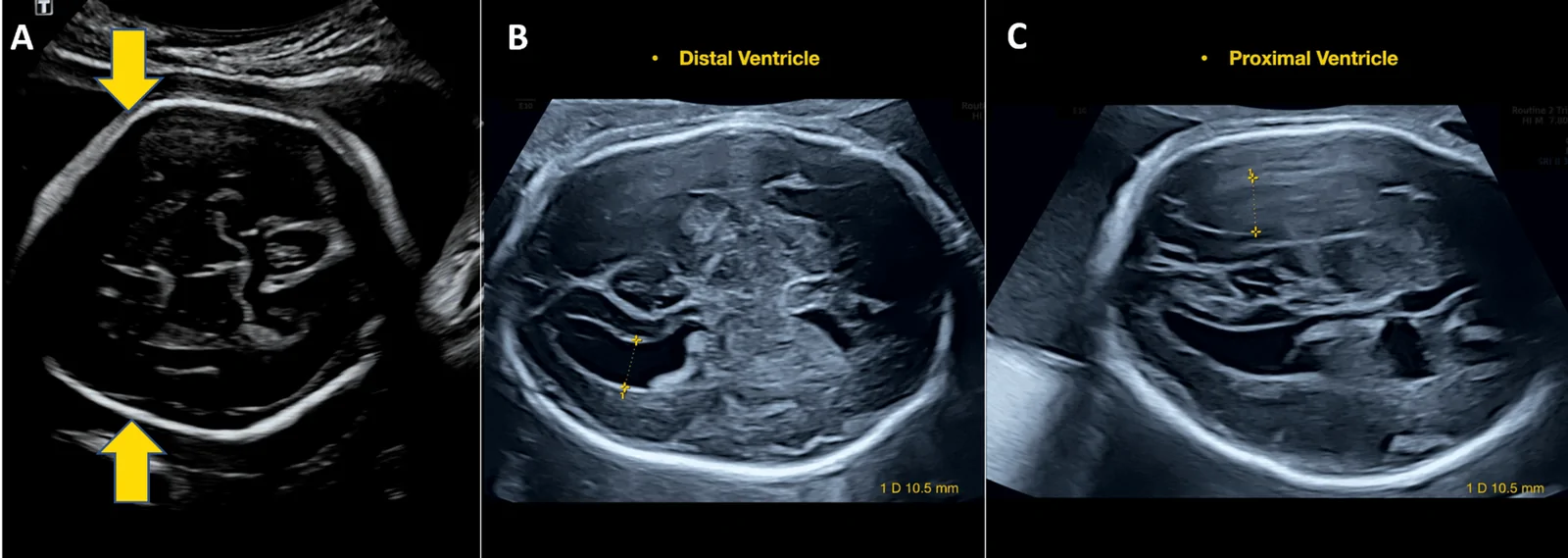
Axial ultrasound of the fetal cranium at 26 5/7 weeks (A) Cranial contour showing the lemon sign (yellow arrows). (B) Labeled distal and (C) proximal transventricular views with inner-to-inner calipers at the atria, documenting bilateral ventriculomegaly (10.5 mm)

**Figure 2:**
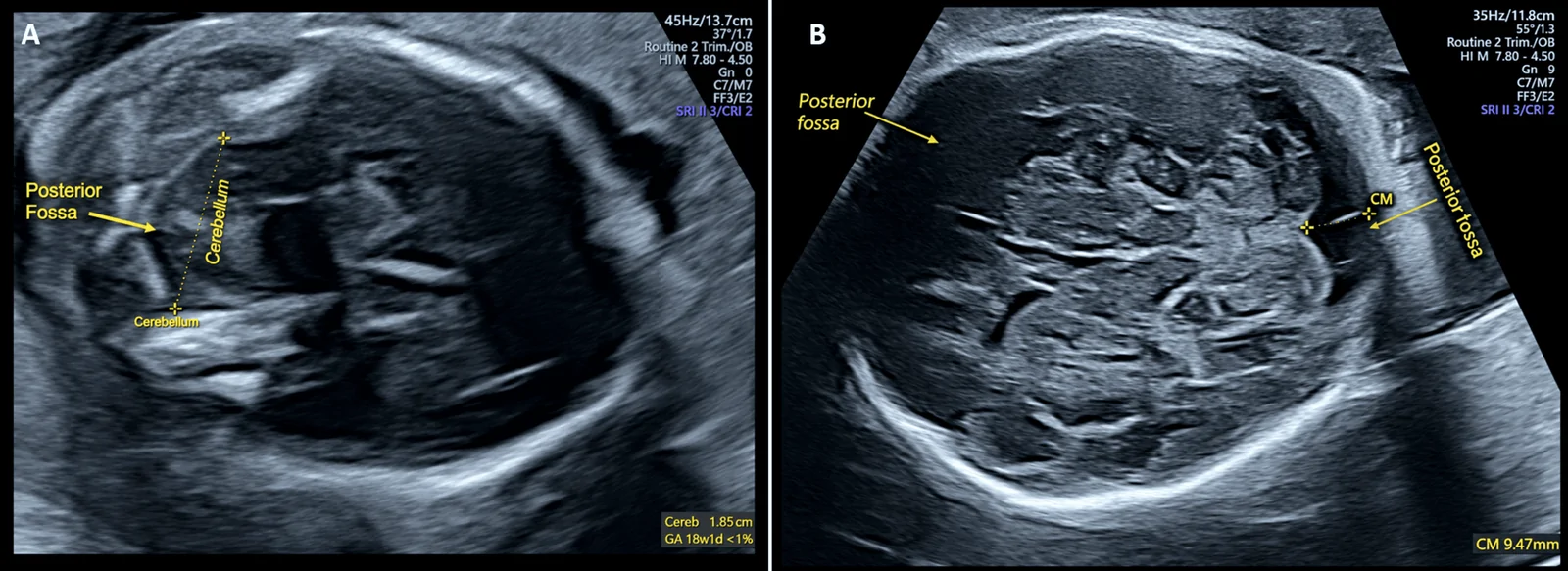
Pre- and postoperative ultrasound views of the fetal posterior fossa demonstrating hindbrain herniation regression (A) Baseline pre-surgical view at 18 1/7 weeks of gestation, showing an obliterated posterior fossa consistent with Arnold-Chiari II malformation. The transverse cerebellar diameter is measured at 1.85 cm. (B) Comparable postoperative view demonstrating restoration of the posterior fossa anatomy, with a visible and measurable cisterna magna (CM) of 9.47 mm

**Figure 3:**
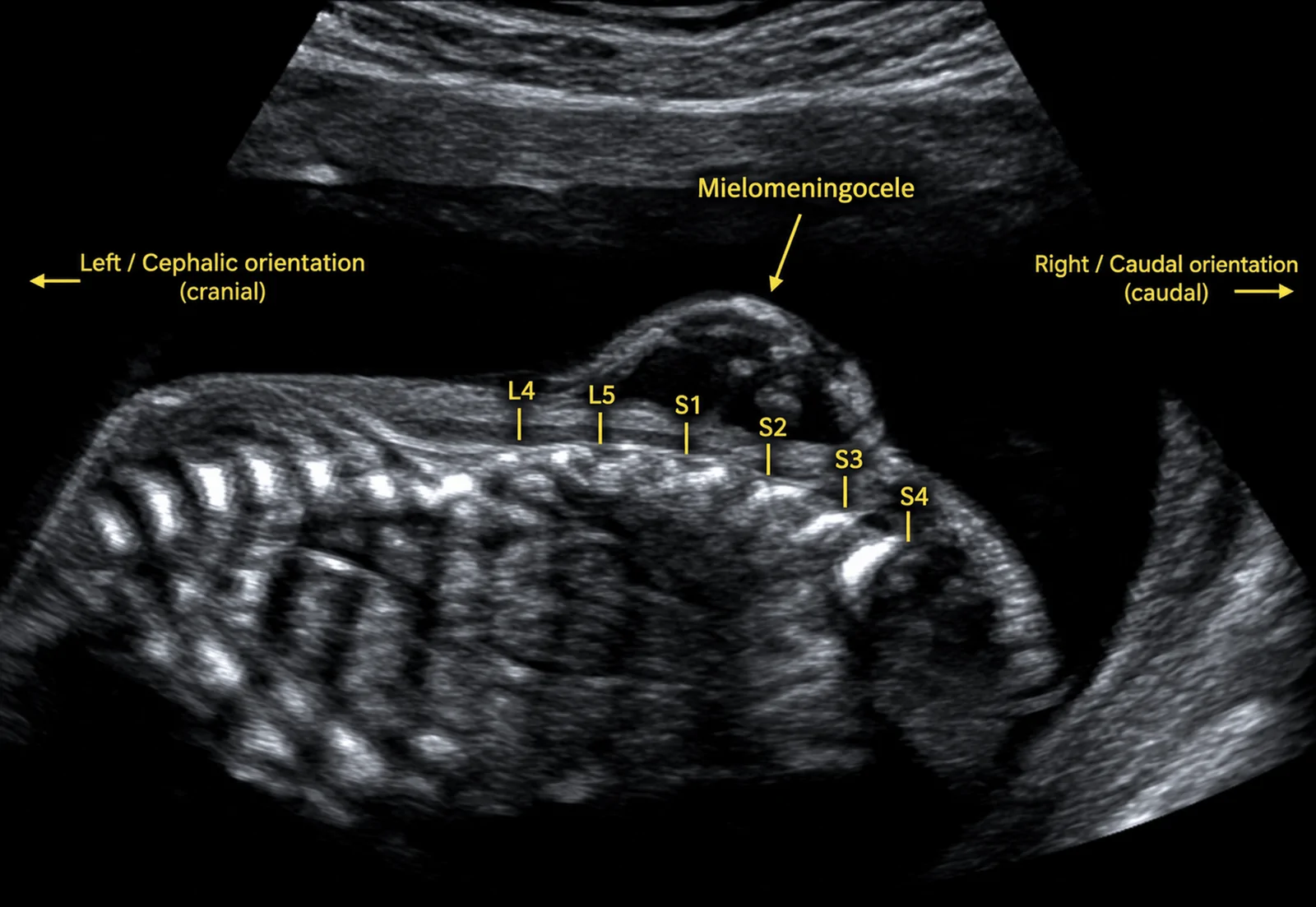
Sagittal ultrasound of the fetal spine demonstrating (yellow arrow) the lumbosacral myelomeningocele at L5-S3

**Figure 4:**
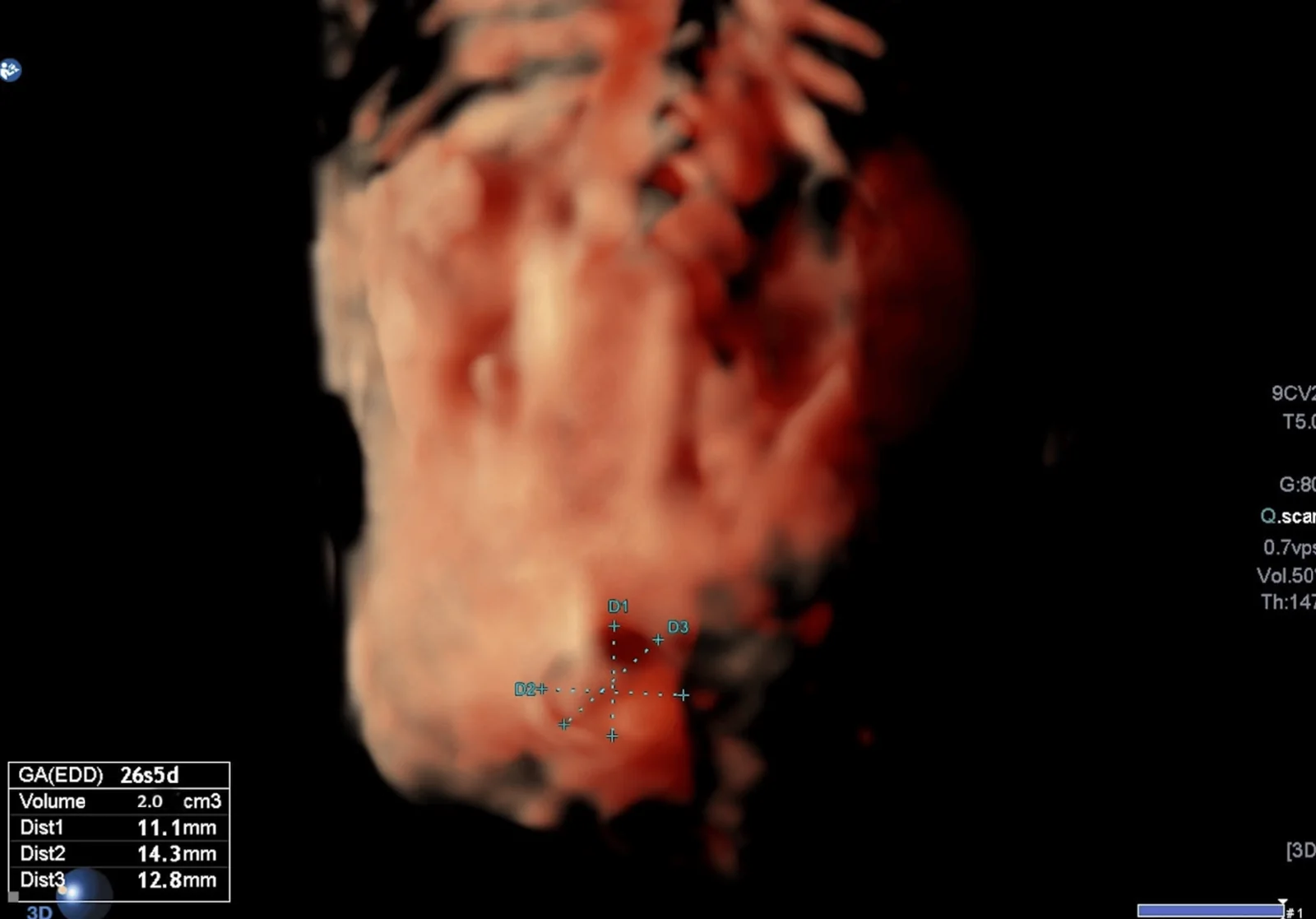
Three-dimensional rendering of the MMC sac at 26 5/7 weeks gestational age The linear dimensions (D1-D3) represent the sac. Orthogonal dimensions are D1 = 11.1 mm, D2 = 14.3 mm, and D3 = 12.8 mm. The estimated volume is 2.0 cm³ by ellipsoid approximation (V = π/6 × D1 × D2 × D3) using the ultrasound system's built-in software MMC: myelomeningocele

Estimated fetal weight was 1,083 g (57th percentile, Hadlock formula). A bicornuate uterus was identified by direct intraoperative inspection, which showed an external fundal indentation of more than 1 cm, consistent with the European Society of Human Reproduction and Embryology and European Society for Gynaecological Endoscopy (ESHRE-ESGE) classification criteria. The fetus occupied the right (gravid) horn. An anterior placenta lateralized to the right was confirmed. Doppler indices and amniotic fluid volume were normal (single deepest pocket 6 cm; cervical length 37 mm). Cardiac anatomy was unremarkable.

Fetal MRI was performed but proved inconclusive because of the 2 to 3 mm slice thickness. The MRI report described a closed defect. However, the multidisciplinary team attributed this discordance to inadequate spatial resolution, noting that the International Society of Ultrasound in Obstetrics and Gynecology (ISUOG) recommends 2 to 5 mm slices depending on the structure of interest, and that thinner slices in this range may result in a lower signal-to-noise ratio and diagnostic uncertainty [8]. The team prioritized the ultrasonographic findings, which unequivocally demonstrated classic open spina bifida signs, including the lemon sign, banana sign, and ventriculomegaly. Preoperative workup showed mild anemia (hemoglobin 10.4 g/dL) with normal white cell count, platelets, creatinine, liver enzymes, and coagulation studies. Toxoplasma IgG was reactive, and IgM was non-reactive (past infection). Rapid plasma reagin (RPR) was non-reactive. Vaginal cultures were negative.

A multidisciplinary conference that included Maternal-Fetal Medicine, Fetal Surgery, Pediatric Neurosurgery, Neonatology, Anesthesiology, Intensive Care, Psychology, Rehabilitation, and Pediatric Neurology determined candidacy for prenatal repair. The team acknowledged that the gestational age of 27 5/7 weeks exceeded the MOMS trial inclusion window (19 0/7 to 25 6/7 weeks) [1]. However, given the late referral, favorable fetal anatomy (functional level S1-S2), adequate amniotic fluid, and the absence of alternative surgical options in the region, the team determined, after an individualized risk-benefit assessment, that the potential benefits of prenatal repair outweighed the risks in this specific clinical scenario. This decision was supported by the Argentine experience reporting acceptable outcomes at extended gestational ages [6]. The patient and her family received detailed counseling and provided separate written informed consent for both the surgical procedure and the publication of this case report with anonymized data.

Open fetal microneurosurgery was performed at 27 5/7 weeks under combined general and epidural anesthesia. Through a midline infraumbilical laparotomy, the uterus was exteriorized, and the placental position was confirmed by intraoperative ultrasound (anterior, right-lateralized, within the gravid horn). The bicornuate uterine anatomy required careful selection of the hysterotomy site to avoid the placenta and the uterine septum. External version was used to position the defect anteriorly. Fetal anesthesia (fentanyl, vecuronium, atropine) was administered intramuscularly under ultrasound guidance.

A 2 cm hysterotomy was performed away from the placenta, and then expanded to 3 cm using a Weitlaner retractor. The membranes were fixed with Vicryl 1-0, and continuous antibiotic irrigation (Hartmann solution with ceftriaxone) was initiated. The neural tube defect was exposed using Deaver retractors (Figure 5). The placode (< 3 cm) was dissected microsurgically. The arachnoid and pial layers were approximated with Prolene 5-0, followed by dural closure and layered skin closure with Prolene 5-0. The hysterotomy was closed in two layers with Vicryl 1-0, and the amniotic fluid was restored with warm saline. Estimated blood loss was 200 mL, and surgical time was 164 minutes. Continuous intraoperative Doppler monitoring with recordings at six predefined time points confirmed stable fetal hemodynamics throughout (Table 1).

**Figure 5:**
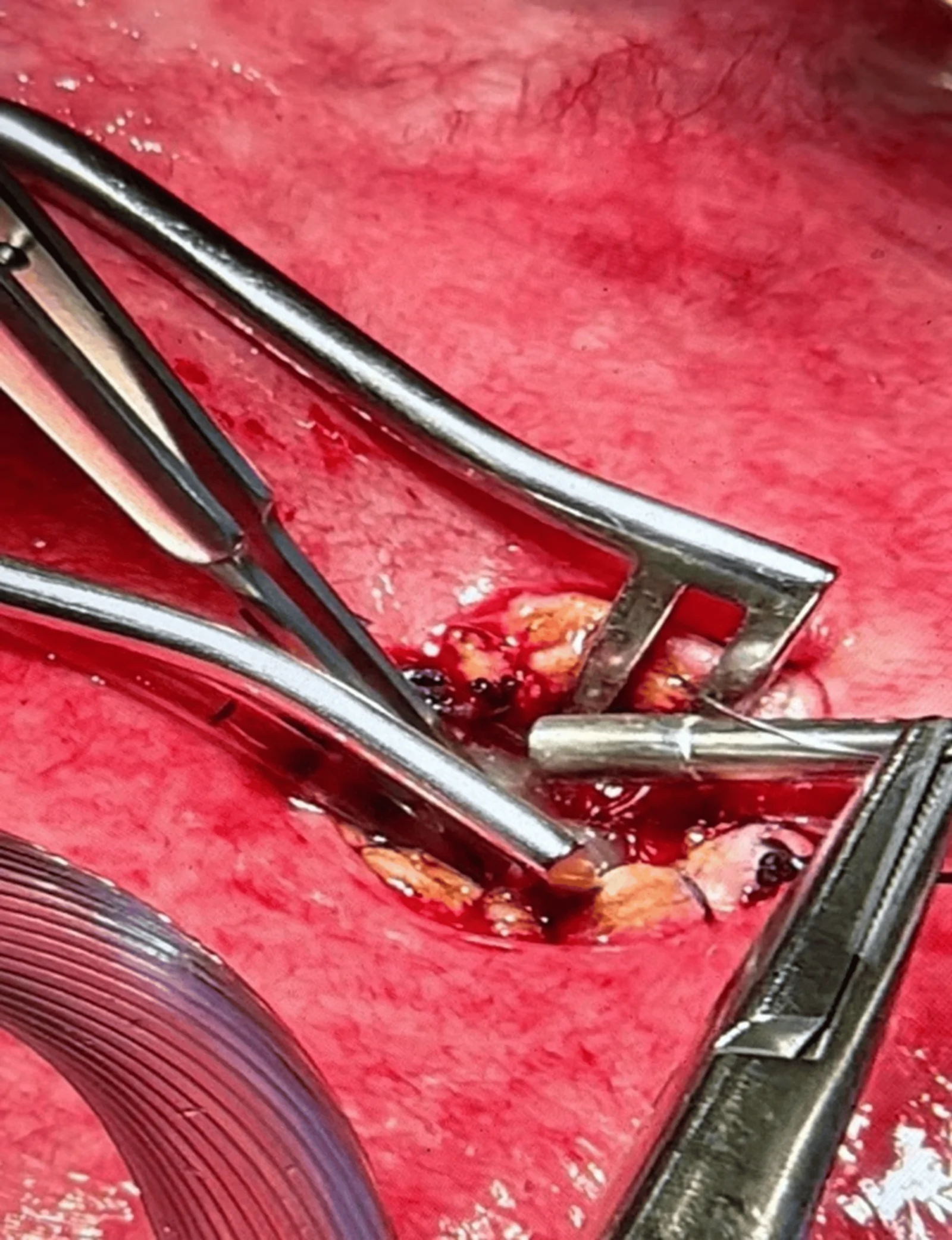
Intraoperative photograph showing the exposed neural tube defect through the hysterotomy during microsurgical repair

**Table 1:** Intraoperative fetal Doppler monitoring Reference ranges at 28 weeks: (UA PI: 0.80-1.20; MCA PI: 1.50-2.30; DV PI: 0.35-0.65 [9]. Doppler measurements were recorded at predefined surgical milestones during continuous monitoring FHR: fetal heart rate; UA PI: umbilical artery pulsatility index; MCA PI: middle cerebral artery pulsatility index; DV PI: ductus venosus pulsatility index

Time point	FHR (bpm)	UA PI	MCA PI	DV PI
Pre-anesthesia	137	1.17	1.58	0.45
Post-induction	143	1.42	1.47	0.45
Pre-exteriorization	122	1.40	1.45	0.45
Pre-myeloplasty	137	1.32	1.20	0.50
Post-myeloplasty	142	1.34	1.27	0.31
Post-reinteriorization	120	1.62	1.90	0.60

The postoperative course was managed in the obstetric ICU. Tocolysis with nifedipine was maintained. Serial ultrasonographic surveillance documented stable bilateral ventriculomegaly (10.5-11.2 mm) with regression of posterior fossa herniation, demonstrated by the restoration of the posterior fossa anatomy and a measurable cisterna magna (Figure 2B). Regression of Chiari malformation was defined as interval increase in the size of the cisterna magna or visible ascent of the cerebellar tonsils above the foramen magnum on serial ultrasound examinations. The repair site remained intact on all follow-up assessments (Table 2).

**Table 2:** Serial postoperative ultrasound follow-up Chiari status assessed by cisterna magna size and cerebellar tonsillar position relative to the foramen magnum on serial ultrasound GA: gestational age; VM: ventriculomegaly; AFI: amniotic fluid index; EFW: estimated fetal weight (Hadlock formula)

Postoperative day	GA (wk)	VM (mm)	AFI (cm)	EFW (g)/percentile	Chiari status
14	30	10.8	12.5	1,520/45th	Partial regression
21	31	11.0	10.2	1,680/48th	Partial regression
35	33	11.2	3.1	2,010/42nd	Stable regression

At 33 weeks, severe oligohydramnios (AFI 3.1 cm) secondary to premature rupture of membranes prompted cesarean delivery through a Munro-Kerr low transverse incision. Inspection of the prior hysterotomy site at the time of delivery revealed no dehiscence of the hysterotomy repair. A male neonate was delivered and weighed 2,060 g, with Apgar scores of 8 at one minute and 9 at five minutes. The lumbar repair site was intact with a well-healed skin closure.

The neonate was admitted to the neonatal ICU (NICU) for eight days. Initial neurological examination revealed a Glasgow Coma Scale (GCS) score of 14, with active and symmetrical spontaneous movements of all extremities. Cranial nerve examination was normal. The anterior fontanelle was normotensive. On lower extremity assessment, spontaneous bilateral hip and knee flexion was present along with active plantar flexion bilaterally, and deep tendon reflexes were normoactive. Anal sphincter tone was present. By NICU discharge, the GCS had improved to 15. Transfontanellar ultrasonography performed during the NICU stay was reported as normal, with no evidence of progressive hydrocephalus. The neonate did not require cerebrospinal fluid (CSF) diversion during the follow-up period. At the last documented evaluation (postnatal day 47), the neurological examination remained stable, with normotensive fontanelles, active lower extremity movements, and an intact lumbar repair site.

## Discussion

This case represents, to our knowledge, the first reported open fetal surgery for MMC in Central America and the first reported prenatal MMC repair performed in a bicornuate uterus with an anterior placenta. Several distinctive features merit discussion. First, the gestational age at intervention (27 5/7 weeks) exceeded the MOMS trial window of 19 0/7 to 25 6/7 weeks [1]. The late referral, a common challenge in LMIC settings with fragmented prenatal care systems, precluded earlier intervention. We considered the Argentine experience reported by Etchegaray et al. [6], which included cases performed beyond 26 weeks with acceptable outcomes, when deliberating on surgical candidacy. In our case, short-term follow-up showed that the repair site remained intact and the neonate did not require CSF diversion during the observation period, although longer follow-up is needed to fully assess outcomes.

Second, the bicornuate uterine anatomy, diagnosed by direct intraoperative inspection demonstrating an external fundal indentation of greater than 1 cm according to ESHRE/ESGE classification criteria, restricted the available hysterotomy sites. The anterior placenta further limited access, requiring precise ultrasound-guided site selection. This anatomical combination has not been previously described in the context of fetal MMC surgery, and its impact on long-term uterine integrity remains to be established. Notably, inspection of the hysterotomy site at the time of cesarean delivery revealed no dehiscence. The patient underwent subtotal hysterectomy during the same operative session, making assessment of future pregnancy uterine integrity not applicable to this individual case.

Third, the discordance between fetal MRI and ultrasonographic findings highlights a relevant diagnostic challenge. The MRI, performed with a 2-3 mm slice thickness, reported a closed defect; the ISUOG Practice Guidelines recommend slice thicknesses of 2-5 mm depending on the anatomical structure of interest, and spatial resolution near the lower end of this range may result in a lower signal-to-noise ratio with consequent diagnostic uncertainty [8]. Rather than representing a fundamental MRI limitation, this discordance more likely reflects the need for optimized MRI protocols in LMIC settings where fetal MRI expertise is still developing. The multidisciplinary team's decision to prioritize ultrasonographic findings was supported by the unequivocal presence of classic signs of open spina bifida.

Fourth, intraoperative Doppler surveillance at six predefined time points, with a seventh measurement obtained after extubation, documented stable fetal hemodynamics throughout the procedure. All values remained within published reference ranges for 28 weeks of gestation [9]. This monitoring strategy, combining continuous Doppler surveillance with systematic recording at predefined surgical milestones, may serve as a feasible and replicable approach in resource-limited settings, although its adequacy cannot be generalized from a single case.

Institutional series from established fetal surgery centers have corroborated the benefits of prenatal repair beyond the original MOMS cohort [10,11], and a systematic review of the decade following the MOMS trial confirmed that both open and fetoscopic techniques continue to demonstrate improvements in maternal and neonatal outcomes [12].

Limitations

This report carries the inherent limitations of a single case. The follow-up period of 47 postnatal days is insufficient to assess long-term neurodevelopmental outcomes, shunt-free survival beyond infancy, or urological function. Genetic testing, which could have identified chromosomal or syndromic etiologies, was unavailable at our institution. The fetal MRI limitations described above reflect institutional constraints rather than a controlled comparison of imaging modalities. The biological rationale for fetal repair, namely, interrupting the progressive spinal cord damage that occurs during gestation, remains compelling and supports continued efforts to expand access to this intervention in underserved regions [13]. Future reports from our center should include prospective longitudinal follow-up with standardized neurodevelopmental assessments.

## Conclusions

Open fetal MMC repair was feasible in an LMIC hospital with appropriate multidisciplinary coordination, even in the presence of a bicornuate uterus with an anterior placenta and at a gestational age beyond the conventional MOMS trial window. During the documented follow-up period, the neonate demonstrated favorable short-term neurological outcomes without the need for CSF diversion. Long-term neurodevelopmental follow-up is essential to confirm the durability of these early results.
